# Blue Sclerae and Differential Diagnosis in Chest Pain: A Case Report

**DOI:** 10.7759/cureus.43542

**Published:** 2023-08-15

**Authors:** Luana C. Miantti Ghellere Bonfim, Isadora S. Guerini, Marjorie G. Zambon, Marcela A. Lopes

**Affiliations:** 1 Department of Medicine, University of South Santa Catarina, Florianopolis, BRA; 2 Department of Medicine, Western Parana State University, Francisco Beltrão, BRA; 3 Department of Medicine, Mackenzie Evangelical University Hospital, Curitiba, BRA; 4 Department of Critical Care, Hospital da Cidade, Salvador, BRA

**Keywords:** chest pain, scoliosis, blue sclerae, costochondritis, skeletal dysplasia, connective tissue disorders, osteogenesis imperfecta

## Abstract

Osteogenesis imperfecta (OI) constitutes a complex connective tissue disorder extending beyond its hallmark bone fragility. This case report explores the intricate diagnostic journey involving an elderly patient with acute chest pain, blue sclerae, and multiple fractures. Despite a thorough cardiac evaluation yielding normal results, the complex medical history and phenotypic markers directed attention toward musculoskeletal factors, underlining the importance of comprehensive diagnostic approaches in hereditary conditions like OI.

## Introduction

Osteogenesis imperfecta (OI), or brittle bone disease, encompasses a genetically and clinically diverse group of inherited connective tissue disorders. The estimated incidence of OI is approximately 1 per 10,000 individuals [1], and about 85-90% are caused by autosomal dominant inheritance [2]. Recent advancements in genetic research have identified over 20 distinct genetic causes, including mutations in type 1 collagen genes, contributing to the phenotypic variations observed in OI. This diversity spans a spectrum of appearances and severity, ranging from mild to severe phenotypes.

A primary characteristic of OI is the presence of low bone mineral density, which predisposes individuals to multiple fractures. However, OI extends beyond bone fragility, affecting the function of various connective tissues, giving rise to numerous combinations of symptoms such as dentinogenesis imperfecta, hearing loss, joint hypermobility, blue sclerae, and basilar invagination, as well as cardiopulmonary defects [3,4].

We present a late-diagnosed OI case associated with the development of costochondritis.

## Case presentation

A 69-year-old female was admitted complaining of burning chest pain radiating to the back, accompanied by severe dyspnea that improved with rest. Her medical history included hypertension, stroke, polio infection, and recurrent bone fractures since childhood. Additionally, a family history of blue sclerae and multiple fractures was noted.

**Figure 1:**
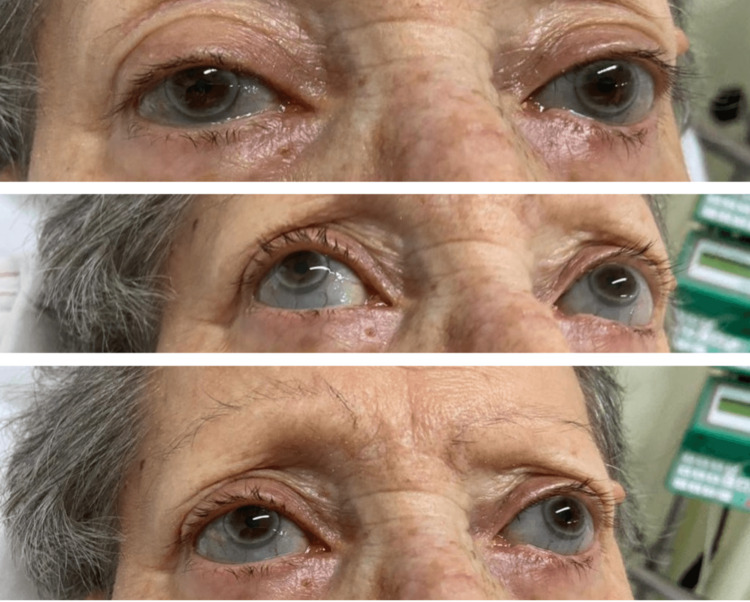
Bilateral bluish sclera

**Figure 2:**
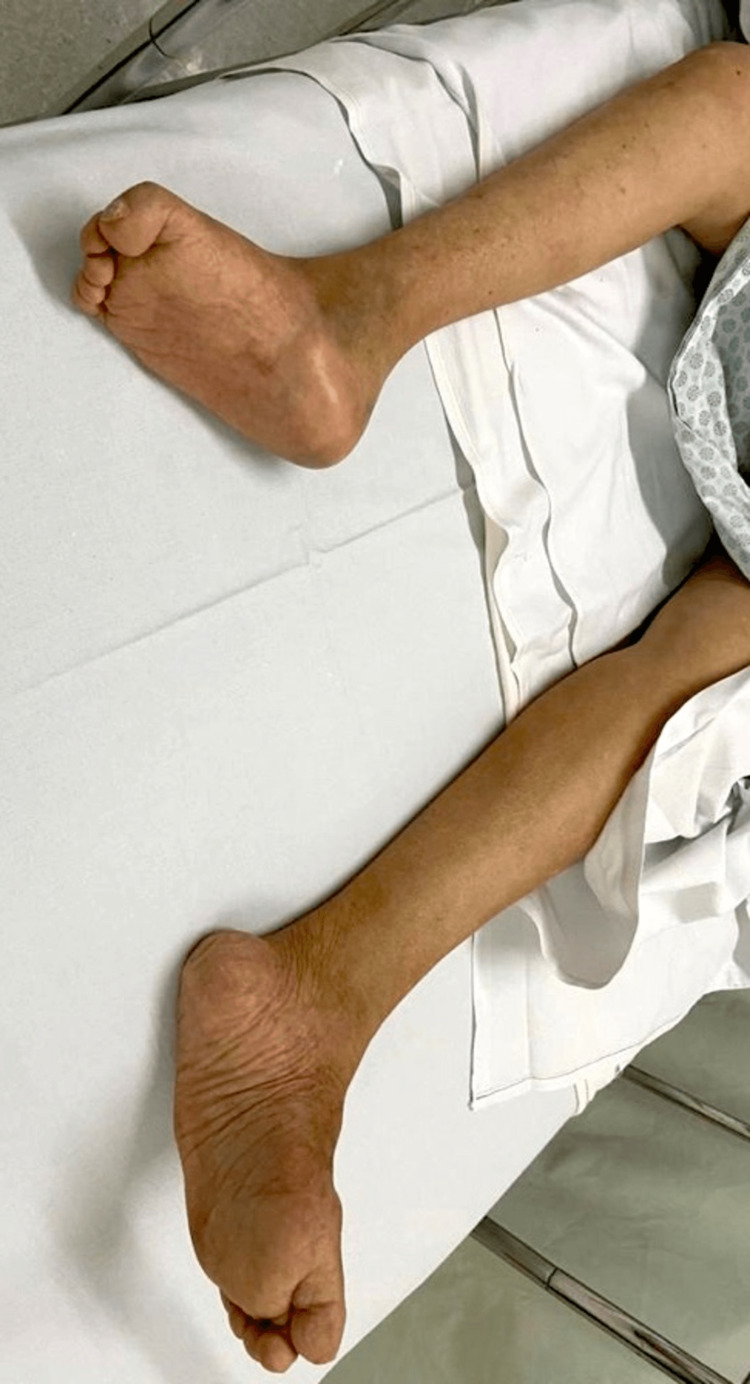
Lower limb deformities

Upon clinical examination, the patient exhibited a series of distinctive features. Progressive hearing loss was evident, along with a visual observation of bluish sclerae (Figure 1). Her physical stature was notably shorter than average, and further examination revealed spinal malformation and deformities in the lower limbs (Figure 2).

**Figure 3:**
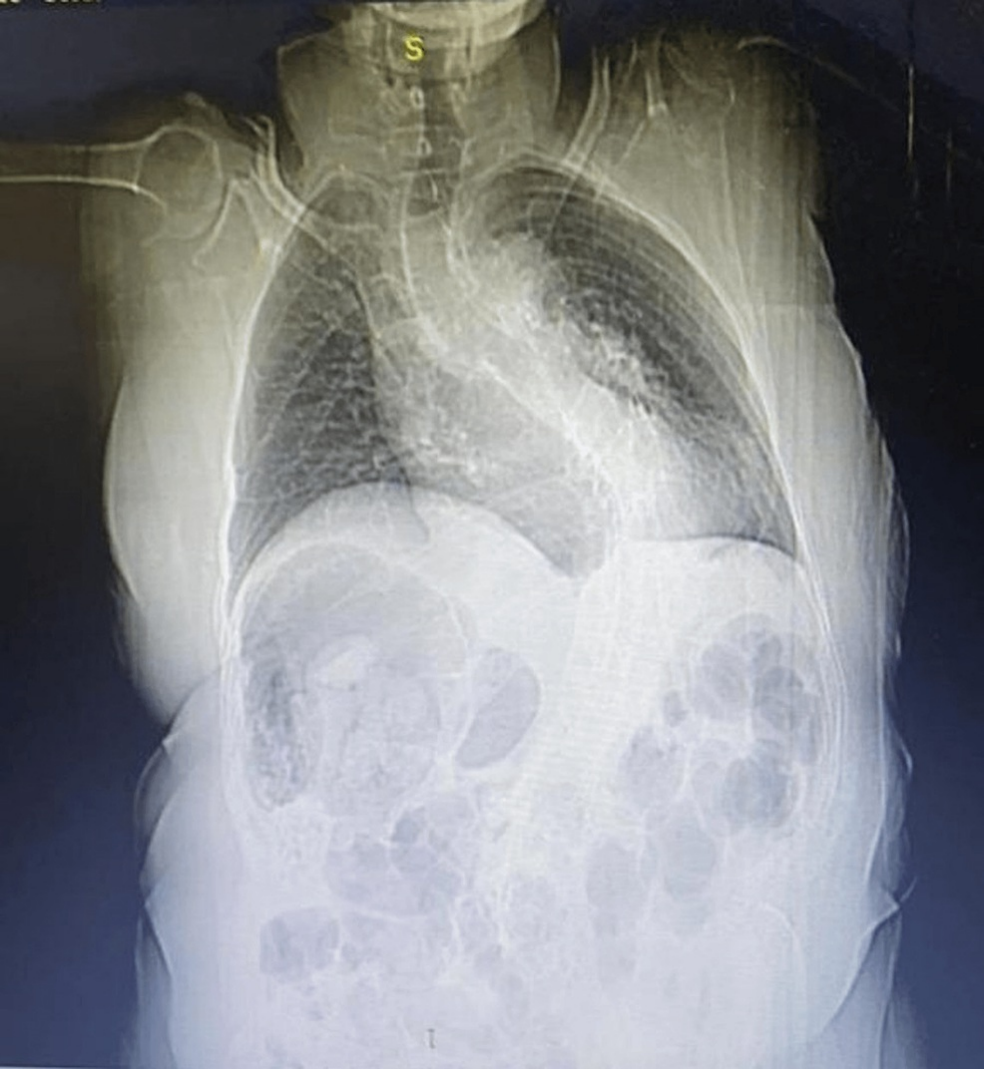
Chest radiograph (CXR) showing severe scoliosis (Cobb angle of ≥50)

Given the nature of the chest pain, a comprehensive evaluation was undertaken to discern its underlying cause. This assessment included an electrocardiogram (ECG) that returned typical results with no indications of cardiac abnormalities. Additionally, cardiac enzyme levels were within the normal range. Echocardiography and coronary angiography were also performed, which yielded unremarkable findings. A chest radiograph (CXR) was conducted and unveiled severe scoliosis (Cobb angle of ≥50) (Figure 3). 

Considering her history, clinical manifestations, and radiographic findings, an OI diagnosis was established. Notably, the patient's chest pain was attributed to costochondritis secondary to OI, owing to chest fractures and an altered thoracic spine associated with skeletal deformities.

## Discussion

Our case presents an intriguing acute chest pain scenario unveiling a distinctive differential diagnosis involving OI and costochondritis. In this context, costochondritis, characterized by inflammation of the costochondral or costosternal joints, emerges as a plausible source of chest pain in OI patients. Despite the patient displaying classical features of OI, the diagnosis was ascertained in later stages, complicating the clinical picture due to the emergence of chronic characteristics.

OI is a systemic connective tissue disorder with inheritance patterns including autosomal dominant, autosomal recessive, or X-linked modes [1,5,6]. Most individuals exhibiting the phenotypic manifestations of OI are attributed to disease-causing dominant variants in the COL1A1 or COL1A2 genes, which encode crucial components of type I collagen. These pathogenic variants can lead to quantitative (haploinsufficiency) or qualitative (structural) collagen defects resulting in mild-to-lethal forms (class I to IV). Although rarer, another dominant form of OI is caused by a recurrent pathogenic variant of the IFITM5 gene, resulting in variable severity (type V). Approximately 15% of cases result from autosomal recessive or X-linked mutations in non-collagen genes responsible for impaired collagen biosynthesis, posttranslational modifications, secretion, and processing or compromised osteoblast function, resulting in moderate-to-severe OI phenotype [5,6]. For many years, defects in collagen genes were believed to be the leading cause of OI, but the range of inheritance modes suggests this disease's heterogeneity [7].

Type I collagen, a primary constituent of various connective tissues, can experience quantity, quality, or related protein perturbations, leading to compromised bone formation and increased fragility. Furthermore, extra-skeletal manifestations can arise due to abnormal bone shape and restricted mobility [1]. Consequently, OI has a broad clinical spectrum in which the phenotypic severity can vary widely, even in identical sequence variants. The 2019 revised Nosology and Classification of Genetic Skeletal Disorders categorizes OI into five distinct types based on clinical characteristics [4]. OI type I is characterized by a mild phenotype, typically presenting with nondeforming limbs and persistently blue sclera. OI type II is associated with perinatal lethality. OI type III is the most severe surviving form related to progressive deformity. OI type IV represents a moderate form, intermediates between OI types I and III, and adults always show normal sclerae. OI type V includes calcification of the interosseous membranes and hypertrophic callus [4].

Among dental and craniofacial clinical manifestations, patients may exhibit dental malocclusion accompanied by dentinogenesis imperfecta [8]. Mild facial dysmorphism or relative macrocephaly might also be present [2]. Ocular problems involve blue sclera, myopia, cornea thinning, trauma, and an increased risk of glaucoma [9]. Hearing loss usually begins in the second to fourth decades of life and is often bilateral and can be conductive, sensorineural, or mixed [10]. While bone fractures decrease after puberty in OI, hearing loss exacerbates over time [10,11].

Fractures are the main characteristics in patients with OI. Patients with OI are more susceptible to recurrent and complex fractures and could have a higher frequency of complications and re-operations [11]. The more severe forms can lead to deformities of long bones, the craniofacial skeleton, the pelvis, and the spine. Common spinal manifestations involve scoliosis, kyphosis, abnormalities in the craniocervical junction, and lumbosacral pathology [12]. Craniocervical junction abnormalities are of particular concern due to potential life-threatening compression of the medulla and cervical spine [13].

Joint hypermobility, often coupled with scoliosis and chest deformities, can affect pulmonary function and stature [11,14]. Cardiovascular complications, including valve insufficiency, heart failure, and vascular aneurysms, tend to manifest in individuals with OI after age 40 [15]. Additional challenges include nonunion fractures, muscle weakness, joint stiffness, and disuse osteopenia [16].

Manifestations of OI in patients can occur at any stage of life, from intrauterine to late adulthood. This diagnostic complexity is evident in our patient's case, necessitating thorough clinical and radiologic evaluation, often over extended periods, to identify mild or atypical presentations [17]. Diagnosis typically involves a blend of clinical and radiologic findings, complemented by genetic testing for confirmation [1]. Advances in imaging technology have enabled early detection of OI during the first or early trimester [18].

In this case, the convergence of the patient's distinctive physical attributes, family history, and recurrent fractures indicated OI. The chest pain, radiating to the back with severe dyspnea, was attributed to secondary costochondritis due to OI. This conclusion was fortified by her history of multiple chest fractures and an altered thoracic spine, correlating with her skeletal deformities. Notably, the absence of abnormal findings during the cardiac assessment, alongside the presence of chronic bone fragility fractures, directed our focus toward musculoskeletal aspects rather than cardiac etiologies for chest pain. Furthermore, given the intricate medical history, we opted to refrain from extensive cardiac testing due to her limited ability to undergo physical stress tests due to her bone disease and the absence of suggestive clinical indications.

While the exact etiology of costochondritis remains uncertain, potential factors like repeated minor chest trauma, rib mobility, scoliosis, osteoarthritis, and scar tissue changes have been posited [19]. Patients commonly report chest pain characterized as either sharp or dull, which can intensify with trunk movements and deep inhalations, yet tends to alleviate with quiet breathing and periods of rest, often accompanied by tenderness upon palpation. Identifying the underlying cause of costochondritis is necessary for appropriate management [20].

No cure exists for OI, and management involves a multidisciplinary approach, including patient education, optimization of bone mass, muscle strength enhancement, and specialized orthopedic care. Bisphosphonates are widely employed to enhance bone density and mitigate fractures [7]. Other therapies, including denosumab, teriparatide, sclerostin inhibition, and TGF-B inhibition, show positive results but still need further studies [16]. Regular hearing assessments are essential since about half of OI patients experience hearing loss after age 40. Consistent surveillance of respiratory, ocular, dental, and cardiac manifestations is imperative across varying severity levels. Addressing functional limitations and chronic pain constitutes an integral aspect of OI management [15].

In this instance, it is evident that our patient did not receive appropriate treatment due to the delayed diagnosis, resulting in the manifestations of chronic features. While several therapeutic approaches are currently under investigation for early-stage OI patients, only a limited number of interventions are directed toward elderly patients [7].

## Conclusions

Our case emphasizes the importance of considering OI in the elderly population. Severe cases are often diagnosed in childhood, while mild cases may not be noticed until later in life. The medical community should consider the differential diagnosis when a young person presents with a history of recurrent fractures and be aware of unusual signs of the disease and family history. Although fracture incidence is lower in adults compared to childhood, the risk of fractures rises again in older age. Timely identification and implementation of appropriate management strategies are pivotal in relieving symptoms and preventing complications for individuals with OI.
